# Patient-specific simulations for planning treatment in congenital heart disease

**DOI:** 10.1098/rsfs.2017.0021

**Published:** 2017-12-15

**Authors:** Claudio Capelli, Emilie Sauvage, Giuliano Giusti, Giorgia M. Bosi, Hopewell Ntsinjana, Mario Carminati, Graham Derrick, Jan Marek, Sachin Khambadkone, Andrew M. Taylor, Silvia Schievano

**Affiliations:** 1 UCL Institute of Cardiovascular Science, London, UK; 2 Great Ormond Street Hospital for Children, NHS Foundation Trust, London, UK; 3 Department of Paediatric Cardiology and Adult Congenital Heart Disease, IRCCS-Policlinico San Donato, San Donato, Milanese, Italy; 4 UCL Department of Mechanical Engineering, London, UK; 5 CH Baragwanath Hospital University of the Witwatersrand, Johannesburg, South Africa

**Keywords:** patient-specific, cardiovascular, congenital heart disease, device, intervention, precision medicine

## Abstract

Patient-specific computational models have been extensively developed over the last decades and applied to investigate a wide range of cardiovascular problems. However, translation of these technologies into clinical applications, such as planning of medical procedures, has been limited to a few single case reports. Hence, the use of patient-specific models is still far from becoming a standard of care in clinical practice. The aim of this study is to describe our experience with a modelling framework that allows patient-specific simulations to be used for prediction of clinical outcomes. A cohort of 12 patients with congenital heart disease who were referred for percutaneous pulmonary valve implantation, stenting of aortic coarctation and surgical repair of double-outlet right ventricle was included in this study. Image data routinely acquired for clinical assessment were post-processed to set up patient-specific models and test device implantation and surgery. Finite-element and computational fluid dynamics analyses were run to assess feasibility of each intervention and provide some guidance. Results showed good agreement between simulations and clinical decision including feasibility, device choice and fluid-dynamic parameters. The promising results of this pilot study support translation of computer simulations as tools for personalization of cardiovascular treatments.

## 1. Background

Over the past two decades, computational models have been extensively developed and adopted to investigate a wide range of cardiovascular problems including cardiac mechanics, haemodynamic conditions and device design [1]. Patient-specific computational tools which combine the advances in the field of clinical imaging and processing, and finite-element (FE) and computational fluid dynamics (CFD) analyses with individual patient data on anatomy and function have greatly supported not only the understanding of human cardiac physiology and pathology, but, by taking into account realistic conditions, also the development of novel interventions and treatments [2,3], thus fostering personalized and precision medicine [4,5]. Bespoke treatment approaches are particularly relevant in the context of congenital heart disease (CHD) as, compared to adult patients with acquired diseases, children born with cardiovascular defects typically present a wide range of different anatomies and conditions that are sometimes unique and extremely complex [6]. Furthermore, with an increasing number of devices available on the market, the choice of the optimal treatment is not always straightforward and the availability of additional predictive tools such as patient-specific computational simulations may become crucial.

However, translation of these computational technologies into clinical practice remains a major challenge for the engineering modelling community [7,8]. In many instances, validation of *in silico* models against *in vivo* results has not been performed on a large scale yet and, if the development of novel tools has attracted large investments, it has not been similarly easy to secure substantial funding and time to test the developed technologies on large numbers of cases. Hence, the use of patient-specific models in CHD is still far from becoming a standard of care and, in the literature, is limited to a few single case reports [9,10].

In the last decade, our engineering group, part of the Cardio-respiratory Unit at Great Ormond Street Hospital for Children, London, UK, has been involved in the development of patient-specific models to support the early introduction of an innovative minimally invasive procedure, the percutaneous pulmonary valve implantation (PPVI) [2,11,12]. From that experience, we have been using computational modelling to study anatomy, physiology and treatments in a wide range of patients with congenital and acquired conditions. This has resulted in the creation of a virtual library [13] with over 1000 anatomical models of cardiovascular structures, including normal subjects and patients with acquired and CHD, and models of devices such as angioplasty balloons, stents, stent-grafts and valved stents. More recently, we have extended the use of such models to address an increasing clinical demand in planning transcatheter interventions and cardiac surgeries in a series of complex CHD cases.

The aim of this work is to report our experience of translating a computer modelling framework into clinics in order to prospectively predict the overall feasibility of specific individual treatments. Patient-specific computational analyses were carried out at the request of the leading clinician for each case in support of the standard decision-making process.

## 2. Methods

The workflow for patient-specific modelling consisted of post-processing of routinely acquired pre-procedural clinical information, including imaging to infer anatomical and functional characteristics of the cardiac structures and great vessels. Simulations were set up in order to mimic cardiovascular procedures and to assess their feasibility by predicting structural and haemodynamic changes, identifying the best matching device for a specific individual implantation site, assessing the device optimal size and positioning, understanding its influence on the local flow field and highlighting potentially compromising spatial interactions with surrounding structures. All simulations were performed prior to the actual treatments, and the results were presented and discussed, along with the patient conventional assessment, at the clinical multi-disciplinary meetings that decide on the most suitable treatment pathway for each individual case.

### 2.1. Patient population

Twelve CHD patients, from six months to 42 years of age (table 1), were referred to our engineering unit from three clinical centres (Great Ormond Street Hospital for Children, London, UK; Baragwanath Hospital University of the Witwatersrand, Johannesburg, South Africa; IRCCS Policlinico San Donato University Hospital, Milan, Italy) with a request to create a patient-specific model to analyse different options of planned procedures as they all presented borderline dimensions or complex anatomical relationships between the different cardiovascular structures for treatment attempts by conventional approaches. Primary diagnosis included tetralogy of Fallot (ToF, *n* = 3), coarctation of the aorta (CoA, *n* = 2), pulmonary atresia (*n* = 2), truncus arteriosus (*n* = 1), hypoplastic left heart syndrome (*n* = 1), double-outlet right ventricle (DORV, *n* = 2) and one case of complex CHD after four open-heart surgeries and two additional thoracic procedures [2]. Associated conditions were ventricular septal defect (VSD, *n* = 3); pulmonary stenosis (*n* = 2); hypoplastic arch (*n* = 2); patent ductus arteriosus (*n* = 2); aberrant right subclavian artery (RSCA, *n* = 1); Ebstein's anomaly (*n* = 1) and MAPCAs (*n* = 1). All patients had undergone previous surgical corrections and/or interventions before referral.

**Table 1.** Demographic data of the patient population.

patient	age (years)	procedure planned	imaging modality	main diagnosis	associated conditions	questions for modelling	modelling technique
Pt01	42	PPVI	CT	complex CHD	multiple procedures	feasibility (new device)	3D, FE
Pt02	17	PPVI	MR	pulmonary atresia	Ebstein's anomaly of the tricuspid valve; RVOT homograft	device choice	3D, FE
Pt03	14	PPVI	MR	tetralogy of Fallot	pulmonary atresia with VSD	feasibility (size)	3D, FE
Pt04	15	PPVI	MR	tetralogy of Fallot	RVOT obstruction	feasibility (size)	3D, FE
Pt05	12	PPVI	MR	truncus arteriosus	surgical repair with Contegra RV-PA conduit	feasibility	3D, FE
Pt06	16	PPVI	MR	pulmonary atresia	VSD, MAPCA, Hancock conduit	feasibility	3D, FE
Pt07	15	PPVI	MR	tetralogy of Fallot	free pulmonary regurgitation, dilated right ventricle	feasibility (compression of the coronaries)	3D, FE
Pt08	19	CoA	CT, MR	coarctation of the aorta	aberrant RSCA in left-sided arch orientation	device size	3D, FE, CFD
Pt09	12	CoA	MR	hypoplastic arch	coarctation of the aorta, aberrant RSCA	device positioning	3D, FE, CFD
Pt010	6	CoA	CT	hypoplastic left heart syndrome	double-outlet RV and hypoplastic arch	device size	3D, FE, CFD
Pt11	0.5	DORV repair	CT	DORV	non-committed VSD, PDA	feasibility (patch size)	3D, CFD
Pt12	0.5	DORV repair	CT	DORV	multiple VSDs, hypoplastic AO ach	feasibility (patch size)	3D, CFD

The treatments planned for the patients were either stenting (*n* = 7 PPVI and *n* = 3 CoA stenting) or surgical procedures (*n* = 2 DORV biventricular repair).

### 2.2. Pre-procedural imaging and processing

Cardiovascular magnetic resonance (MR) images were available in eight cases (Siemens Avanto, 1.5T; GE Medical Signa Excite), all including a three-dimensional (3D) whole-heart sequence (free-breathing and isotropic [14]) that allowed assessment of anatomy and relationship between different morphological structures, 2D cine images across the implantation site to infer information on dynamics and overall material properties and 2D phase contrast images to assess flow [15]. Computed tomography (CT) was performed in five cases—one patient had both CT and MR investigations—using three different systems (Siemens Somatom Force; Siemens Somatom Definition; SA Toshiba Aquilion): in one of these cases [2], four-dimensional (4D) CT was acquired to capture information on ventricular function and implantation site dynamic. Three-dimensional image resolution varied from 0.27 mm (CT) to 1.75 mm (MR).

Additionally, in the three CoA cases, echocardiography images were used to complement the haemodynamic information acquired from MR and CT.

**Figure 1.**
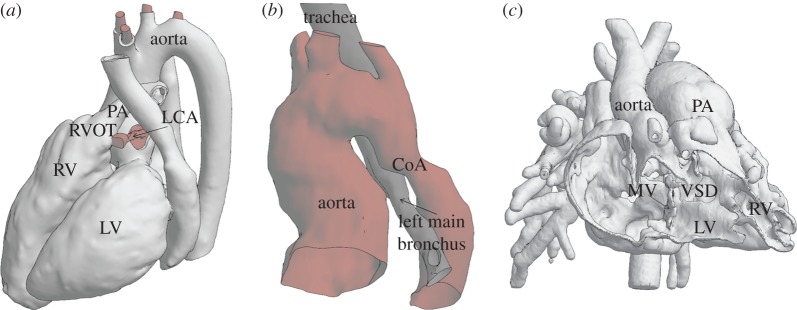
Examples of three patient-specific models included in this study for investigation of anatomical spatial relationships: (*a*) Pt07 referred for PPVI, whole-heart reconstruction including left ventricle (LV), right ventricle (RV), RVOT, pulmonary arteries (PA), left coronary artery (LCA) and aorta; (*b*) 3D model of Pt10 referred for stenting of aortic coarctation including aorta, CoA, trachea and left bronchus; and (*c*) Pt12, frontal section of the DORV heart including mitral valve (MV), LV, RV, great vessels (aorta and PA) and VSD. (Online version in colour.)

Patient images were segmented to create the *in silico* patient-specific model using a combination of automatic and manual tools [16,17] including both commercial and open source software: Mimics (Materialise, Belgium), Simpleware ScanIp (Synopsis, UK), VMTKLab (Orobix, Italy), ITK-SNAP (www.itksnap.org [18]) and OsiriX (Pixmeo, Switzerland). Three-dimensional anatomical volumes of the blood pool were created from the 3D whole-heart MR sequences and from CT in order to represent the region of interest for the treatment and the surrounding structures which may affect or be affected by device implantation or surgical correction: to simulate PPVI, the right ventricular outflow tract (RVOT), pulmonary trunk and the proximal branch of the pulmonary arteries were modelled together with the aortic root and coronary arteries (Pt07, in figure 1*a*). To model CoA stenting, ascending aorta, aortic arch and descending aorta were reconstructed including the branch vessels; the reconstruction of the bronchi was added in one case (Pt10, in figure 1*b*). To simulate DORV surgical repair, models of intracardiac anatomies were reconstructed with both right and left ventricles, cardiac valves and origin of the great vessels (Pt12, in figure 1*c*). Further refinement of the surfaces and preparation of the anatomies for simulation, for example, the creation of inlet and outlet planar surfaces, were achieved using MeshMixer (Autodesk, USA [19]). The patches manufactured in DORV repair were designed (Rhinoceros, McNeel) to replicate the intraventricular tunnel from the VSD to the semilunar systemic valve, in order to connect the left ventricle to the systemic circulation and restore physiological circulation.

MR 2D cine images coupled with cardiovascular pressure information were used to derive overall material properties, while patient-specific boundary conditions for CFD analysis were extracted from MR 2D phase contrast images and echocardiography.

### 2.3. Patient-specific simulations

The simulation methodologies included FE analyses which were performed with the commercial software Abaqus/Explicit (Dessault Systemes) and CFD analyses carried out with the commercial packages Fluent (Ansys) and VMTKLab (Orobix). PPVI and CoA stenting were modelled by virtually deploying devices within the patient-specific reconstructed surface anatomies. The 3D surfaces were meshed with shell elements with size ranging from 0.3 to 1.8 mm following sensitivity analysis and matching the dimensions and complexity of each model. Materials of the cardiovascular structures were modelled with linear elastic characteristics: the Young's modulus of the different patient-specific models varied between 42 and 700 kPa according to the distensibility of the structures assessed from imaging [20], and Poisson's ratio was set to 0.45.

For the catheterization procedures, the models of the devices resembled the balloon-expandable Melody TPV^®^ (Medtronic, USA), Sapien TPV (Edwards Lifesciences LLC, USA), CP™ covered stent (NuMED, USA) and the novel self-expandable PPVI device [2] (Medtronic), including multiple sizes and deployment balloons when necessary. Design and material characteristics of the devices are summarized in table 2. For each intervention, multiple simulations were run by modelling different positioning, opening configurations/diameters or exploring the combination of multiple devices.

**Table 2.** Characteristics of the devices modelled for the virtual interventions.

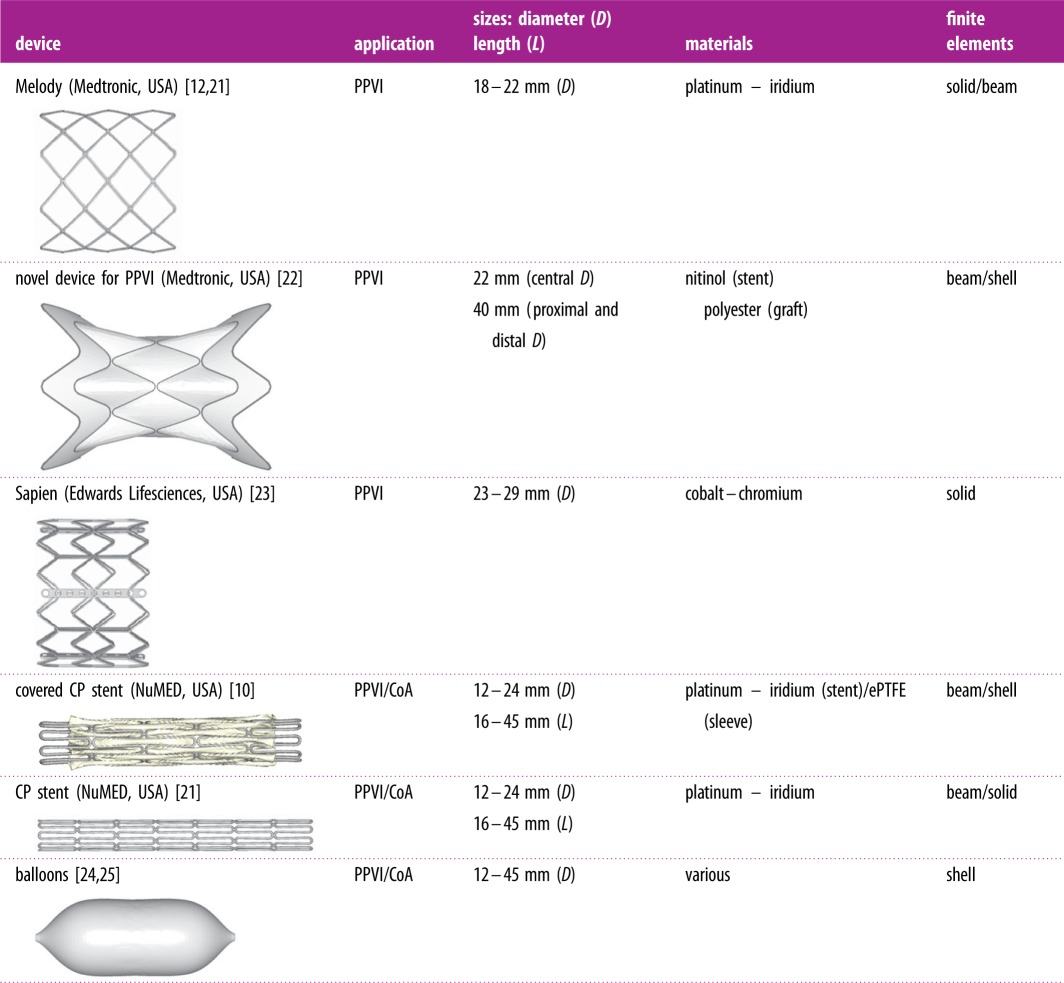

CoA cases were additionally analysed using CFD to evaluate the patient-specific changes of haemodynamics (i.e. variation of pressure gradient and velocity) due to the interventions. CFD simulations were also set up to explore the potential haemodynamics following the surgical repair of DORVs. The 3D surface reconstructed from pre-procedural images and the CoA post-stenting anatomies extracted from the FE simulations were filled up with 3D elements to model the blood pool. Blood was modelled as a viscoelastic fluid (density 1.025 kg m^−13^; viscosity 0.0035 kg/(m s)). A default *κ*–*ω* SST turbulence model was taken into account in the simulations of the CoA performed with Fluent. Patient-specific boundary conditions at inlets and outlets were extracted from flow data measured by MR and echocardiography.

The parameters of interest from the FE and CFD analyses were the deformed configurations of the vessels, contact areas between devices and implantation sites (in terms of distributions of stresses on the vessel wall) and flow data such as velocity distribution and pressure fields. All these results were provided to the clinicians in the form of visual representation of potential post-implantation scenarios.

### 2.4. Simulated versus clinical treatment

The primary evaluation of the computer modelling pipeline was based on the agreement with the feasibility of each intervention. The indications predicted by the simulations and the actual clinical decisions were therefore compared for each patient case. Intra- and post-procedural data and images collected during intervention and follow-up were used to estimate the level of accuracy of the simulations. For the PPVI cases, post-implantation fluoroscopy images were post-processed to reconstruct the 3D geometry of the stent *in situ* and measure the size of the expanded stent. In addition, FE results were superimposed to the post-implantation fluoroscopy projections to assess device positioning. For the CoA cases, fluoroscopy images were used to assess the size of the stent, while post-implantation pressure and flow from catheterization measurements, ultrasound and MR imaging, when available, were compared to the residual gradient and fluid dynamics calculated with CFD. Finally, for DORV surgical repair, post-operative echocardiography and CT images were used to assess anatomy and function of the intracardiac tunnel.

## 3. Results

**Figure 2.**
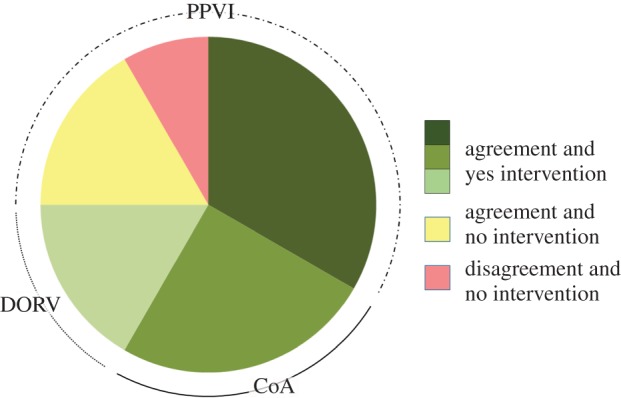
The pie chart summarizes the agreement between simulations and procedural decisions of the cases included in this study: in nine cases (green), there was agreement between simulation results and clinical decisions, and the planned intervention was performed; in two cases (yellow), there was agreement between simulation results and clinical decisions, and the planned intervention was not performed; in one case (red), there was disagreement between simulation results and clinical decisions, and the planned intervention was not performed. (Online version in colour.)

Each engineering analysis was completed prior to the actual procedure with no requirements for additional clinical data. After discussion at the multi-disciplinary meeting, all 12 patients underwent successful catheterization or surgery according to the, respectively, planned treatment before modelling and with no intra-operative complications reported. The results of the simulations and the main clinical outcomes are reported in the following paragraphs, grouped according to the modelled treatment. Agreement with the indications rising from the computer simulations is summarized in figure 2.

Details of the simulated cases are here grouped according to the procedure.

### 3.1. Percutaneous pulmonary valve implantation

In five patients (Pt01, Pt02, Pt05, Pt06, Pt07) with borderline outflow tract characteristics, the FE PPVI procedures suggested feasibility of the intervention with deployment of Melody stent in three cases, Sapien 29 and the new device for PPVI in one case each. Feasibility was guaranteed in the simulation by the final diameter of the expanded stents, which was in accordance with the guidelines for each device (i.e. max 22 mm for Melody stent; max 29 mm for Sapien stent).

**Figure 3.**
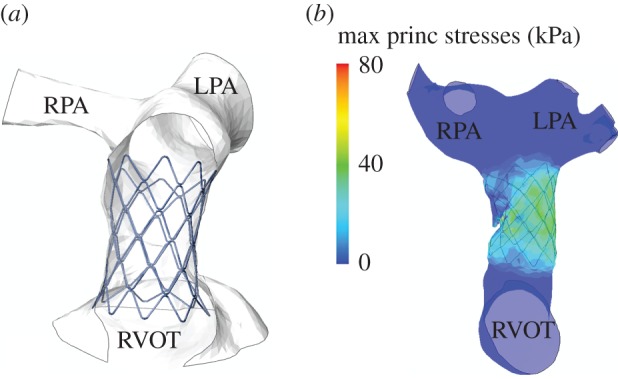
Two simulations of feasible PPVI: (*a*) deformed configuration of Pt05 with Melody stent virtually implanted (frontal view cut); and (*b*) stress distribution following the implant of the stent in Pt06 confirming safe anchoring. (Online version in colour.)

**Figure 4.**
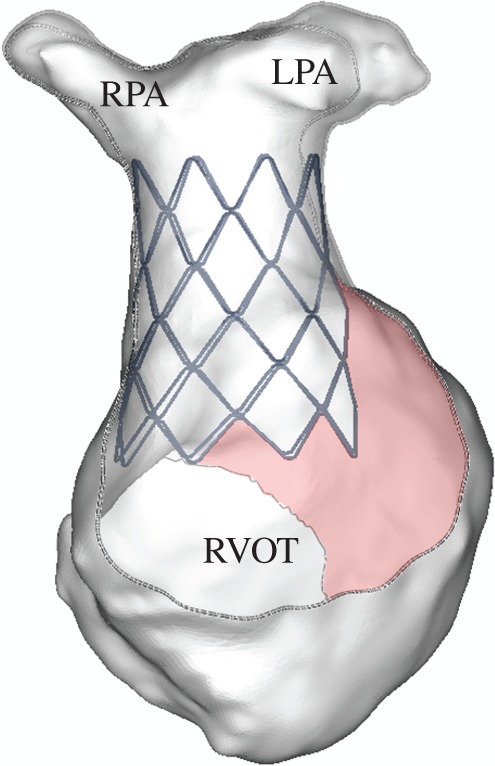
Frontal view cut of the deformed configuration of Pt03 highlighting lack of contact in the proximal part of the stent (pink areas). (Online version in colour.)

The analysis of the stent/implantation site interaction [20] confirmed that the cross-sections of the stent were in full contact with the arterial wall, suggesting safe anchoring of the device (deployed configuration and stress distribution are shown in figure 3), but also no evidence of interference with surrounding structures such as compression of the coronary arteries. The two remaining cases were considered not suitable for PPVI because in one case, a large portion of the stent was found not in full contact with the wall, thus indicating no anchoring in the proximal part of the stent for Pt03 (figure 4) and, in the second case, the coronary artery of Pt04 was found to be compressed by the expansion of the stent.

PPVI was accomplished successfully and according to the FE indications in four patients. In one case (Pt02), PPVI was not performed due to high distensibility of the implantation size after sizing balloon and high risk of device embolization. In Pt03, during the catheterization procedure, the size of the proximal part of the RVOT was tested with a sizing balloon and verified to be too large to allow safe anchoring. Hence, PPVI was not performed. In Pt04, a non-compliant balloon was inflated within the RVOT at the same size as planned for the PPVI device and simultaneous selective coronary angiography demonstrated significant compression of the left coronary artery. Hence, PPVI was not completed and the patient was referred for open-heart surgery.

### 3.2. Coarctation of the aorta

**Figure 5.**
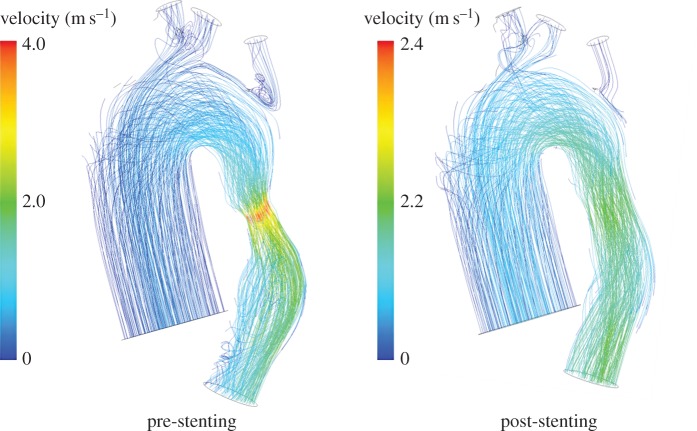
Comparison of the velocity streamlines for the CoA of Pt10 before and after the virtual implantation of a stent to relieve the restriction of the descending aorta. Within the post-stenting geometry, the peak velocity decreases and the downstream flow is less disturbed. (Online version in colour.)

The FE simulations identified the maximum expansion diameter allowed for the covered stent at the level of the narrowing in the descending aorta in order to avoid obstruction of the origin of the aberrant RSCA in Pt08 (16 mm) and Pt09 (12 mm) and compression of the bronchi in Pt10 (12 mm). The CFD analyses quantified a decrease both in the peak velocity (e.g. Pt10 in figure 5) and pressure gradient which in all virtually treated CoA dropped from an average of 15.5 mmHg pre-implant to 1.9 mmHg after stenting.

In the catheterization laboratory, CoA stenting was performed successfully in all three cases and according to the indications provided by the FE simulations in terms of sizing and positioning, resulting in a reduction in the gradient across the coarctation to less than 1 mmHg.

### 3.3. Double-outlet right ventricle

**Figure 6.**
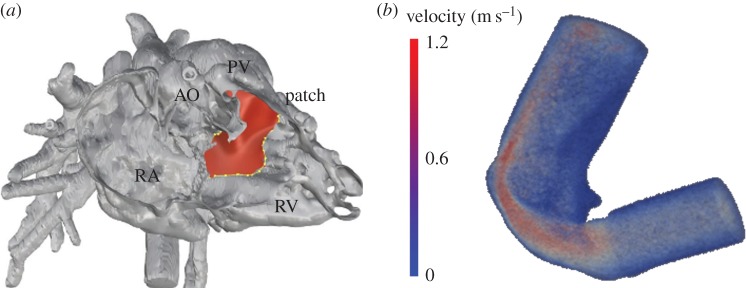
Model of DORV case Pt11: (*a*) the virtual surgery shows the design of the patch (red) aiming to create the baffle between the ventricle and the aorta; and (*b*) velocity field result of the CFD analyses performed on the new virtual geometry. (Online version in colour.)

The tunnel geometries resulting from the virtual surgery on Pt11 (figure 6*a*) and Pt12 had a minimum diameter of 6.9 and 6.0 mm, respectively. The ratio of the cross-sectional areas of the baffle and aortic annulus were 1.07 and 0.81, respectively. CFD analyses carried out on the treated virtual surgery geometries showed a peak velocity of 1.1 m s^−1^ for Pt11 (figure 6*b*) and 1.9 m s^−1^ for Pt12, at the narrowest section of the virtual baffle. The analysis of the tunnel geometrical parameters and CFD results suggested the feasibility of a morphological biventricular repair pathway with acceptable haemodynamics for these patients. This was achieved in surgery, with no significant gradient measured at echocardiography during follow-up.

**Figure 7.**
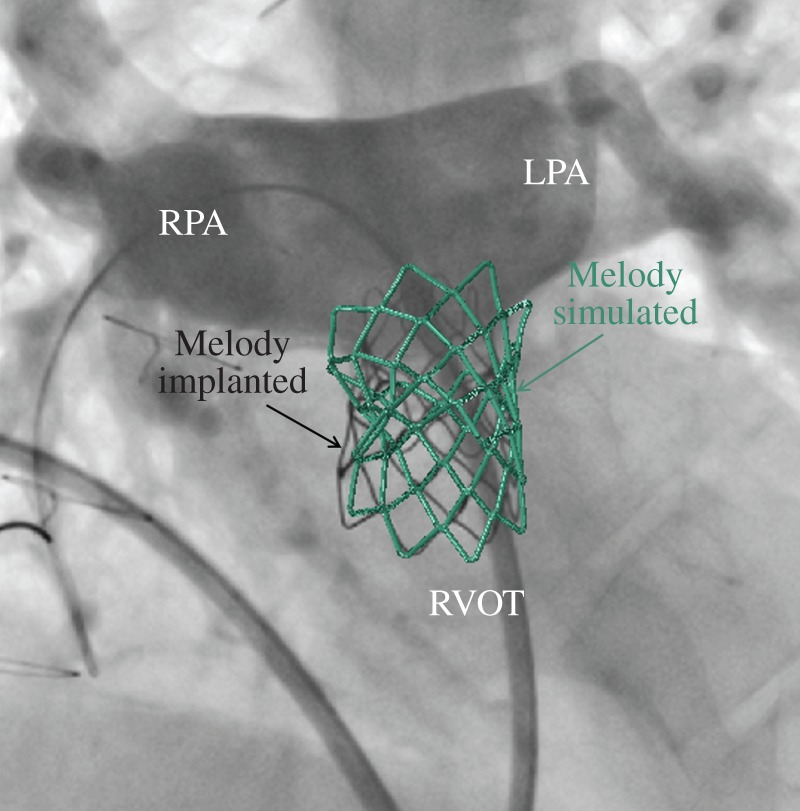
Superimposition of the simulated device on the intraprocedural fluoroscopy images for the PPVI case Pt06. (Online version in colour.)

**Figure 8.**
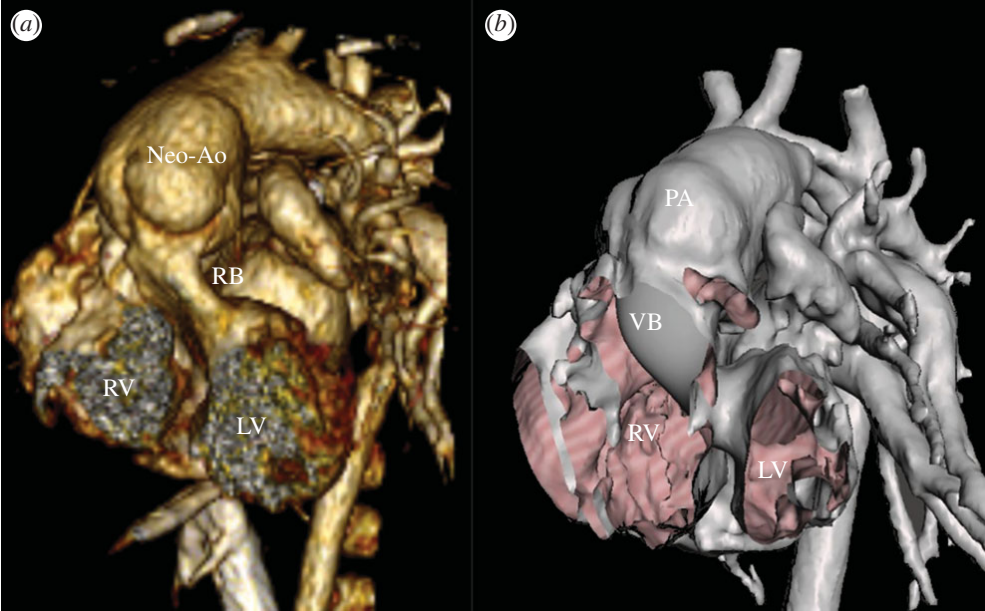
Comparison of geometries following surgical biventricular repair of DORV case Pt11: (*a*) post-procedural CT reconstruction highlighting right ventricle (RV), left ventricle (LV), neo-aorta (Neo-Ao) and real baffle (RB); and (*b*) virtual geometry including RV, LV, pulmonary artery (PA) and virtual baffle (VB). The PA was not switched to Neo-Ao in the model. (Online version in colour.)

In summary, the predictions of the computational analyses were in accordance with the delivered treatment in all cases included in this study except in one case of PPVI (figure 2). When devices were implanted, the post-procedural fluoroscopy images were superimposed to the models resulting from the computational simulations (Pt06 in figure 7) and confirmed correct sizing and positioning of the stent in PPVI and CoA cases with an average difference in the stent sizes of 1.2 and 0.8 mm, respectively. Pressure and velocity data acquired by transthoracic echocardiography showed excellent agreement with the results calculated with CFD analyses for CoA stenting and DORV repairs with a max error less than 3 mmHg. One of the DORV patients had a CT post-intervention: 3D reconstruction showed a tunnel geometry similar to the shape virtually created before the surgery (Pt11 in figure 8), with a difference in the minimum diameter of 0.5 mm.

## 4. Discussion

This study reports our early experience in prospective use of patient-specific computational modelling to support the decision-making process for complex CHD cases and explores the translation of patient-specific simulations into clinical practice. FE and CFD analyses were applied to predict the post-operative scenarios of specific procedures, including minimally invasive interventions such as implantation of stents for pulmonary valve replacement and aortic coarctation relieve, and complex surgical treatments such as DORV biventricular repair.

The complexity of the cases included in this study advocated the use of patient-specific computational modelling. In fact, patients were all born with complex CHD, and presented uniquely different anatomies and physiologies requiring full understanding of the spatial relations between cardiovascular structures and of the delicate balance in function. In this context, personalized computer simulations can explore multiple treatment options for each individual clinical case, highlighting advantages and disadvantages of each approach, with no harm for the patient. They could help identify the optimal management whether this is a surgical operation or the implantation of a device or the testing of a non-standard novel solution.

The results on this small cohort of patients are promising as simulations were able to predict the feasibility of treatments and optimize delivery parameters in agreement with the clinical decisions and delivered treatments in all cases, but one PPVI procedure. The disagreement between computer prediction and clinical decision in this case was related to the distensibility of the implantation site which did not recommend the implantation of a PPVI device [20] following invasive assessment. This highlighted the need for further studies and new methodologies for reverse engineering and inferring patient-specific mechanical properties. Indeed, while over the last decades, cardiovascular imaging has shown remarkable advances in morphology acquisition, it remains problematic to infer a complete description of the material properties (i.e. beyond the physiological range of deformation) by means of non-invasive methods.

Out of the 12 analysed cases, nine patients received treatment in the form of stenting or surgical procedure: in these, the simulations were able to predict the post-operative scenario with a remarkable level of correspondence in terms of measurements on the devices and haemodynamics. In those two patients where the simulations suggested unsuitable conditions for PPVI stenting, the catheterization procedure was carried out anyway, and balloon testing performed before valved stent implantation confirmed the results of the simulations. If computer simulations could be fully trusted, procedures could sometimes be avoided.

To build clinicians’ confidence in engineering modelling, patient-specific simulations in CHD have to be challenged and validated in larger clinical studies. A conventional randomized clinical trial to test the effect of inclusion of modelling in the decision process in the context of complex CHD may be difficult, as numbers are limited and each patient condition is often unique. However, as a starting point, in this work, we have shown a small series of cases in which the validation is suggested as based mainly on agreement/disagreement with clinical decision and comparison with real immediate post-procedural results. While some discrepancy in terms of positioning/dimensions or local haemodynamics in the comparison with real data after the procedure may be clinically acceptable, it is the validity of the agreement/disagreement on feasibility that need to be further tested together with additional information and benefits that the simulation can provide. On these grounds, the criteria of acceptance of simulation results would need to be further defined by a larger consensus and the validity of this method should be further tested on larger populations of patients. In this context, it would be important to explore different computational modelling techniques to assess not only immediate, but also mid- and long-term results of different procedures in each patient condition.

Initial translation of computational modelling in clinical practice has been possible only as a result of a multi-disciplinary approach, implying continuous interaction between engineers and clinicians. The computational methods adopted in this study were validated from an engineering point of view (i.e. *in silico* versus *in vitro*) and multiple simulation strategies and tools were used to answer different questions, with no unique workflow. Therefore, technical expertise is required but, importantly, our experience has shown that the computational approach can work remotely, on cases referred from clinical centres from different countries.

The pilot nature of this study indicates that there are margins for improvement, as already identified. First of all, boundary conditions for patient-specific simulations based uniquely on routinely acquired clinical data and non-invasive images as in this study need to be improved to account for the variability encountered in this patient population. Hence, the impact of this limitation will need to be investigated by means of statistical studies which can take into account the level of uncertainties [26]. Such analysis will need to consider other sources of uncertainties including the choices of algorithms, physical properties and operator dependency. Second, the series here reported is small, and more cases and more conditions need to be simulated in order to provide stronger clinical evidence on the usefulness of prospective computational models. Third, the influence of computational tools on the actual decision-making process has not been evaluated yet. Further studies, purposely designed, will be required to evaluate the impact that simulations might have on the different phases of the cognitive process, including comprehension of the problem, review of possible solutions, definition of decision criteria and, finally, selection of the most satisfying solutions. Finally, a rigorous cost analysis has not been performed. While the general opinion is that computational analyses are inexpensive, a refined examination which quantifies direct and indirect costs should be carried out in order to assess more precisely the cost/benefit ratio.

Nevertheless, clinical translation of computer simulations for CHD has started and it is realistic to foresee more and more applications in this direction in the coming years. This will contribute to fulfil the original mission of the VPH which aimed to personalize care for each specific patient and to improve it by integrating computer precision into cardiovascular medicine.

## 5. Conclusion

This study reports the early results of a prospective study which aimed to translate patient-specific computer modelling into clinical practice for the treatment of complex CHD. Simulation results showed agreement with clinical decision to assess the feasibility of the procedures in greater than 90% of the analysed cases. Patient-specific simulations can support the decision-making process by providing guidance for the management of complex cases of CHD.

## Data accessibility

This article has no additional data.
